# A Novel Insight on Signal Transduction Mechanism of RcsCDB System in *Salmonella enterica* Serovar Typhimurium

**DOI:** 10.1371/journal.pone.0072527

**Published:** 2013-09-04

**Authors:** María de las Mercedes Pescaretti, Juan V. Farizano, Roberto Morero, Mónica A. Delgado

**Affiliations:** Instituto Superior de Investigaciones Biológicas (Consejo Nacional de Investigaciones Científicas y Técnicas-Universidad Nacional de Tucumán) and Instituto de Química Biológica “Dr. Bernabe Bloj”, Tucumán, Argentina; Institut National de la Recherche Agronomique, France

## Abstract

The RcsCDB system of *Salmonella enterica* serovar Typhimurium is implicated in the control of capsule and flagella synthesis. The hybrid sensor RcsC, the phosphotransferase RcsD and the RcsB regulator, constitute the main components of the RcsCDB system. The proposed Rcs signaling cascade involves the autophosphorylation of RcsC and the transfer of the phosphate group to RcsB, mediated by RcsD. We previously reported that the overexpression of *rcsB* repress the transcription of *rcsD* by an autoregulation mechanism. Moreover, we demonstrated that during the *rcsD* repression, the RcsB-dependent flagellar modulation remained active. These results suggest that the Rcs phosphorelay mechanism occurs even in the absence of RcsD. In this work, we established the existence of two alternative phosphorelay pathways driving activation of this system. We demonstrated that RcsC and RcsD can act as histidine kinase proteins which, after autophosphorylated, are able to independently transfer the phosphate to RcsB. Our results suggest that these pathways could be activated by different environmental signals, leading different levels of RcsB-phosphorylated to produce a differential gene modulation. These findings contribute to a better understanding of the complexity and importance of the Rcs system activation, where more than one phosphate flow pathway increases the possibilities to exert gene regulation for a quick environmental changes response.

## Introduction

The RcsCDB phosphorelay system is unique and most likely presents a conserved structure and features among different bacterial species. This is widely used by bacteria to monitor environmental changes, converting specific external signals in changes of gene expression that is required for adaptation to new conditions. The RcsCDB is a complex unorthodox signaling system composed by the transmembrane sensor kinase RcsC, the response regulator RcsB and the RcsD transmembrane protein considered as the phosphorelay intermediary between RcsC and RcsB [1]. In this regard, the phosphate-transfer mechanism involve several phosphate binding domains in these proteins containing a conserved H or D amino acid residues by which travels the phosphate group [1]. The presence of a particular stimulus produces the autophosphorylation of the RcsC histidine-kinase domain (HK) involving the H1 (His 479). Afterward, the phosphate group passes, into the same protein, to D1 of the receiver domain (Asp 875) [2]. To conserve the phosphate-transfer pattern, the consecutive phosphorylation occurs in H2 of the RcsD histidine phosphotransfer domain (HPt), which finally transfers this phosphate to the RcsB-D2 receiver domain [1], [3].

The Rcs phosphorelay system participates in the response to extracytoplasmic stress signaling that affect the cell envelope. The current knowledge of the Rcs activation conditions involve the exposition to polymyxin B [4], [5], the bacteria growth at low temperature or on solid surface [6], mutations of *tolB* gene, the grown of *pmrA* mutants in presence of iron, the punctual mutation of *rcsC* namely *rcsC11* constitutive mutant [7], [8], the *rcsB* overexpression [9], between others. Despite of this wide variety of conditions, the specific environmental signal able to activate the Rcs system has yet to be determined.

Although the system was initially identified as the mechanism that controls the *cps* operon expression, required for biosynthesis of the capsular polysaccharide colanic acid [10], it has been also implicated in the control of flagellar genes [11], and biofilm formation [12], as well as in other virulence processes [5], [13]–[15].

In *Salmonella enterica* serovar Typhimurium (*S.* Typhimurium), it has been previously demonstrated that high levels of RcsB can modulate Rcs-dependent promoters, some of which are controlled in an RcsC-independent pathway [9], [16]. Moreover, we reported that the overexpression of *rcsB* gene lead to a negative autoregulation mechanism by repression of the *rcsD* transcription [17]. However, we observed that under this state the modulation of some RcsB-dependent genes, like the *flhDC* operon expression, were maintained [17]. Based on these data we ask us: why in this multi-step phosphorelay system one of its components is negatively regulated under full activation condition, and why the activity of some RcsB-dependent promoters can be modulated in the absence of the RcsC sensor. A possible explanation is that there could be additional signal transduction mechanisms to the previously described by Takeda *et al.* [3]. In this work we studied whether RcsC and RcsD have the ability to autophosphorylate and to transfer the phosphate group to their cognate regulator. We here examined for the first time the specificity of Rcs system component interactions necessary to drive the mentioned functions, using *in vivo* and *in vitro* assays. We established that RcsC and RcsD act as independent kinase proteins able to drive the RcsB phosphorylation to control the expression of those genes required to respond at different conditions. Taken together, our results demonstrated that the RcsCDB system activation can be carried out at least by three different phosphate flow mechanisms, two of them here described. These findings increase the knowledge of the RcsCDB system to understand why this system is activated under several conditions.

## Materials and Methods

### Bacterial Strains and Growth Conditions

The bacterial strains and plasmids used in this study are shown in Table 1. The mutations were introduced into the bacterial chromosome by P22-mediated transductions as described [18]. The recombinant DNA methods were performed using the standard protocols [19]. *S.* Typhimurium and *Escherichia coli* (*E. coli*) were grown in Luria–Bertani (LB) at 30 or 37°C as noted. Antibiotics were used at the following final concentrations: 50 µg kanamycin/ml, 50 µg ampicillin/ml and 25 µg chloramphenicol/ml. To measure activity of *cps*::MudJ or *flhDC*::MudJ transcriptional fusions by *rcsB* gene overexpression, bacteria were grown to an OD_600_ = 0.2 (approximately 2 h). Then, 0.35 mM IPTG (final concentration) was added to induce the overexpression of *rcsB* from the P*_lac_* promoter of p*rcsB* plasmid. After 3 h of growth, β-galactosidase activity was determined as described below. A negative control assay was carried out using the p*rcsB*op plasmid harboring the *rcsB* gene cloned in opposite direction to the *lacZY* promoter. Moreover, the effect of osmotic shock by sucrose (0.464 M final concentration) was assayed to measure the *cps*::MudJ and *flhDC*::MudJ expression under other Rcs system activation, using the protocol previously described [20].

**Table 1:** Bacterial strains and plasmids used in this study.

Strain or plasmid	Description^a^	Reference or source
***S.*** ** Typhimurium strains**		
14028s	wild-type	Fields *et al*. (1986) [37]
EG13384	*cps*::MudJ	Mouslim and Groisman (2003) [38]
EG13772	*flhDC5213*::MudJ	Mouslim *et al*. (2003) [39]
EG14498	Δ*rcsC*::Cm	Pescaretti *et al.* (2009)
MDs1117	Δ*rcsD*::Cm	This work
MDs1574	*cps*::MudJ Δ*rcsD*::Cm	This work
MDs1575	*cps*::MudJ Δ*rcsC*::Cm	This work
MDs1576	*cps*::MudJ Δ*rcsD* Δ*rcsC*::Cm	This work
MDs1577	*flhDC5213*::MudJ Δ*rcsD*::Cm	This work
MDs1578	*flhDC5213*::MudJ Δ*rcsC*::Cm	This work
MDs1579	*flhDC5213*::MudJ Δ*rcsD* Δ*rcsC*::Cm	This work
MDs1557	*cps*::MudJ *ackA*::Tn*10*Tc	This work
MDs1558	*cps*::MudJ *ackA*::Tn*10*Tc Δ*rcsC*::Cm	This work
MDs1559	*cps*::MudJ *ackA*::Tn*10*Tc Δ*rcsD*::Cm	This work
MDs1562	*cps*::MudJ *ackA*::Tn*10*Tc Δ*rcsD*::FRT Δ*rcsC*::Cm	This work
***E. coli*** ** strains**		
DHM1	F-, *cya-854*, *recA1, endA1, gyrA96 (Nal), thi1, hsdR17,spoT1, rfbD1, glnV44(AS).*	Karimova *et al*. (2005)
MDs1265	BL21 DE3 Δ*rcsDBC*::Cm	This work
***Plasmids***		
pUHE2-2*lacI^q^*	rep_pMB1_ Ap^r^ *lacI^q^*	Soncini *et al*. (1995) [40]
p*rcsB*	pUHE2-21 *lacI^q^* containing *rcsB* gene	Pescaretti *et al.* (2009)
p*rcsB*op	pUHE2-21 *lacI^q^* containing *rcsB* gene in opposite direction of the lac promoter	Pescaretti *et al.* (2009)
pT7-7	rep_PMB1_ Ap^r^ pT7	Tabor and Richardson (1985)
pT7-7-rcsB-His6	rep_PMB1_ Ap^r^ pT7-*rcsB*-His6	Delgado *et al.* (2006)
pT*rcsC*cytHT	rep_PMB1_ Ap^r^ pT7-*rcsC*cyt-His6	This work
pT*rcsD*cytHT	rep_PMB1_ Ap^r^ pT7-*rcsD*cyt-His6	This work
pUT18	Ap^r^, Col E1 *ori*, vector for fusion to N-terminus of Cya-T18	Karimova *et al.* (1998)
pUT18C	Ap^r^, Col E1 *ori*, vector for fusion to C-terminus of Cya-T18	Karimova *et al.* (1998)
pKT25	Km^r^, p15A *ori*, vector for fusion to C-terminus of Cya-T25	Karimova *et al.* (1998)
pKTN25	Km^r^, p15A *ori*, vector for fusion to N-terminus of Cya-T25	Karimova *et al.* (1998)
pUT18C-Zip	pUT18 with the leucine zipper domain of the yeast GCN4 activator	Karimova *et al.* (1998)
pKT25-Zip	pKT25 containing leucine zipper domain of the yeast GCN4 activator	Karimova *et al.* (1998)
pKD3	*bla* FRT *cat* FRT PS1 PS2 oriR6K	Datsenko and Wanner (2000)
pKD46	*bla* P_BAD_ *gam bet exo* pSC101 oriTS	Datsenko and Wanner (2000)

Gene designations are summarized by Sanderson *et al.* (1995) [41]. Nal, nalidixic acid resistance; Ap, ampicillin resistance; Km, kanamycin resistance; Cm, Cloramphenicol resistance.

### Mutants and Plasmid Constructions

The chromosomal *rcsD* gene mutation of wild-type *S.* Typhimurium 14028s strain and the deletion of the region containing the *rcsD*, *rcsB* and *rcsC* genes from the *E. coli* BL21 DE3 strain chromosome were performed using the one-step gene inactivation method [21]. In brief, the *rcsD* and *rcsDBC* mutations were constructed using primers 2385/4504 and 4083/4084 (Table 2), to amplify the chloramphenicol resistance cassette (Cm) from pKD3 plasmid. The deletion mutations were confirmed by direct nucleotide sequencing. When was necessary the resistance cassette was removed using the plasmid pCP20 as previously described [21].

**Table 2:** Primers used in this work.

(#)	Name	5′ to 3′ sequence
1133	Fwd *rcsB* BACTH	CGG GAT CCC AAC AAT ATG AAC GTA ATT ATT G
2385	Fwd Δ*rcsD*	GCG TTG CTT TTA CAG GTC GTA AAC ATA ATG TAG GCT GGA GCT GCT TC
4504	Rev Δ*rcsD*	CGT TTC ACA TAA CTG CTT GCC GGG TAC CAG ATT AAG CAT GGC CAT ATG AAT ATC CTC CTT AG
4083	Fw Δ*rcsDBC*	CAC ACT GTA CCC TTT ATA CTG CCC TAT CAC TTC GCG AAG TGT GTA GGC TGG AGC TGC TTC
4084	Rev Δ*rcsDBC*	GCT TCG CCC CTT TGA AAT ACC TTG CTT CTT TTC GTA CCA TAT GAA TAT CCT CCT TAG
4201	Rev *rcsC*cyt	CGG GAT CCT TAT GCC CGC GTT TTA CGT ACC C
4202	Rev *rcsD*cyt	CGG GAT CCC TAC AGC AAG CTT TTG ACG TAG GCG
4228	Fwd *rcsC*cyt	GGA ATT CCA TAT GCA CCA CCA CCA CCA CCA CGC GCG GAT GTA TGA GCG GCG
4229	Fwd *rcsD*cyt	GGA ATT CCA TAT GCA CCA CCA CCA CCA CCA CCG TCA TCA GCC GGG ACG GTC G
8006	Rev *rcsC* BACTH	CGG GGT ACC GGT GCC CGC GTT TTA CGT ACC CGC TCG GC
8007	Fwd *rcsC*cyt BACTH	CGC GGA TCC CCT TGC GCG GAT GTA TGA GCG GCG
8010	Rev *rcsD* BACTH	CTA GTC TAG ACA GCA AGC TTT TGA CGT AGG CGT CAA TGT C
8011	Rev *rcsB* BACTH	CGG GGT ACC GGT TCT TTG TCT GTC GGA CTC
8022	Fwd *rcsD*cyt BACTH	CGC GGA TCC CCA TCA GCC GGG ACG GTC GAC G

Plasmids for overproduction of RcsCcyt or RcsDcyt were constructed by amplification of DNA fragments containing the coding sequence of the RcsC or RcsD cytoplasmic domain, including nucleotides 999 to 2847 and 990 to 2670, respectively. In this assay we used the *S.* Typhimurium 14028s genome as template and primers 4228/4201 for RcsCcyt or 4229/4202 for RcsDcyt, containing the BamHI/HindIII restriction sites (Table 2). After digestion, the DNA fragments were cloned into the corresponding restriction sites of pT7-7 vector [22]. A His_6_-tag was also added in the N-terminus of each coding sequence to facilitate the purification using Ni^2+^-NTA affinity chromatography.

### Bacterial Two-hybrid (BACTH) Assay

Studies of protein-protein interaction were performed as described by Karimova *et al.* [23]. The BACTH system is based in the functional complementation of T25 and T18 fragments of the adenylate cyclase enzyme to rely the cAMP and activates the *lacZY* genes transcription. The coding sequence of the cytoplasmic domain of RcsC and RcsD, and the full-length of RcsB were cloned *in frame* with T18 or T25 adenylate cyclase fragments, using the BACTH plasmids pUT18, pUT18C, pKT25 and pKTN25. To the convenient cloning into the BACTH plasmids, the BamHI/KpnI restriction sites were introduced into the primers for *rcsC*cyt and *rcsB*, while the BamHI/XbaI was used for *rcsD*cyt (Table 2). The correct *in-frame* junction sites were confirmed by DNA sequencing. The *E. coli* DHM1 strain was then co-transformed in all possible combinations with the BACTH derivatives plasmids (Table 1). Then, the co-transformed strains were streaked on LB plates containing the appropriated antibiotics and incubated for approximately 3 days at 30°C. Following the Karimova *et al.* [23] protocol, the obtained colonies were used to inoculate LB medium supplemented with antibiotics and 0.5 mM IPTG (final concentration) for plasmid selection and proteins expression, respectively. The cultures were grown for 12 h at 30°C and then these cultures were used to determine the β-galactosidase activity as described below. The *E. coli* DHM1 harboring the empty vectors pUT18 and pKT25 was used as negative proteins interaction, while *E. coli* DHM1 co-transformed with pKT25-zip and pUT18C-zip plasmids, containing the leucine zippers GCN4 domain, was used as highest proteins interaction. In addition, unspecific interactions were discarded in a new control experiments carried out using pT25-*rcsC*, -*rcsD* or -*rcsB* cotransformed with empty pT18 or using pT18-*rcsC*, -*rcsD* or -*rcsB* cotransformed with empty pT25.

### β-galactosidase Assays

The β-galactosidase activity was determined at room temperature in permeabilized cells as previously described [24].

### Proteins Overexpression and Purification

To overexpress the proteins, 100 ml of LB medium were inoculated with *E. coli* BL21 DE3 *rcsDBC*::Cm mutant containing pT*rcsC*cyt-His_6_ or pT*rcsD*cyt-His_6_ and incubated at 37°C for 2 h to reach an OD_600nm_ = 0.3–0.4. The expression of the His-tagged RcsCcyt and RcsDcyt proteins was induced with 0.1 mM IPTG and the cultures were further incubated at 30°C for 3 h (OD_600 nm_ = 0.6). Cells were harvested by centrifugation at 8,000 g for 20 min at 4°C, and stored at −70°C overnight. To protein purification, the frozen cells were resuspended in 10 ml of lysis buffer (300 mM NaCl; 30 mM NaH_2_PO_4_ pH 8.3; 15 mM imidazole) containing 100 mM phenylmethylsulfonyl fluoride, and disrupted in a French pressure at 18,000 psi. Cellular debris was removed by centrifugation at 13,000 g, for 40 min at 4°C. The soluble fraction containing the proteins was mixed in batch with 2 ml of 50% Ni^2+^-NTA agarose resin (Qiagen), previously equilibrated with lysis buffer, and incubated for 3 h at 4°C to allow the binding of protein to the resin. After centrifugation, the resin containing proteins was washed 3 times with lysis buffer containing increasing concentration of imidazole (30, 50 or 100 mM, respectively). Finally, the His-tagged RcsCcyt and RcsDcyt proteins were eluted from the Ni-NTA resin using 300 mM imidazole. The purified proteins samples were supplemented with glycerol (50%, v/v) and stored at −70°C. The purity of the samples was confirmed by electrophoresis in SDS-PAGE. The RcsB-His_6_ and PmrA-His_6_ proteins used in this work were purified as described [25].

### Determination of Protein Concentration

The protein concentration was determined using the protein BCA kit (PIERCE) as described [26].

### Phosphorylation Assays

The autophosphorylation assay was performed as described [27]. Briefly, 5 µM RcsCcyt-His_6_ or RcsDcyt-His_6_ were incubated at room temperature with 3.75 µCi [γ-^32^P]-ATP (3000 Ci/mmole) in 30 µl of TBS/1 mM MgCl_2_/1 mM DTT. Phosphorylation was stopped by addition of 4× SDS sample buffer. The phosphorylated proteins were analyzed by SDS-PAGE 12% (w/v). Gels were dried and autoradiographed.

The phosphotransfer assay was performed as described [27]. In brief, 30 µl of [γ-^32^P]-RcsCcyt-His_6_ or [γ-^32^P]-RcsDcyt-His_6_ were mixed with 30 µl of TBS/1 mM MgCl_2_/1 mM DTT containing 10 µM RcsB or PmrA (as negative control). The reaction was stopped at different times by adding 4× SDS sample buffer. The phosphorylated proteins were separated by SDS-PAGE 12% (w/v). Gels were dried and autoradiographed.

## Results

### The Rcs Signaling by *rcsB* Overexpression Requires the Presence of RcsC or RcsD

**Figure 1:**
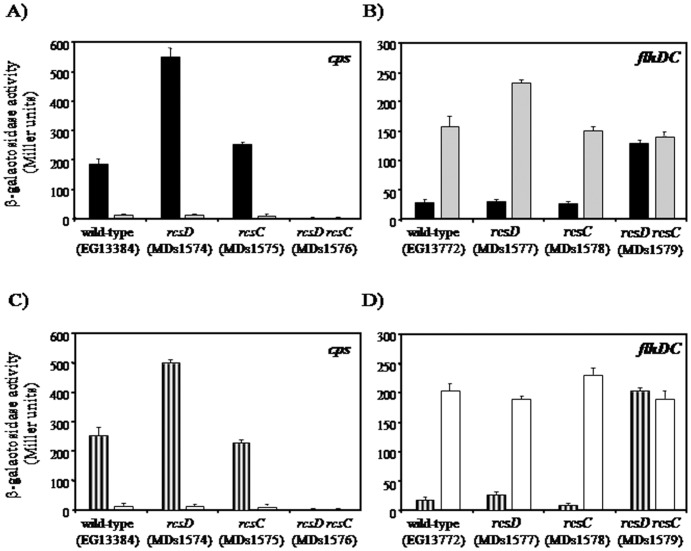
Modulation of *cps* and *flhDC* operons by the RcsC- or RcsD-dependent presence. The β-galactosidase activity (Miller units), produced by *rcsB* overexpression, from *cps*::MudJ (**A**) and *flhDC*::MudJ (**B**) *lac* transcriptional fusions was investigated in the following genetic backgrounds: wild-type, *rcsD*, *rcsC* and *rcsD rcsC*, harboring the p*rcsB* (black bars) or p*rcsB*op (grey bars) plasmids. These strains were grown at 37°C in LB medium supplemented with 0.35 mM IPTG. The osmotic shock effect by sucrose addition (striped bars) on *cps*::MudJ (**C**) and *flhDC*::MudJ (D) expression was investigated in the wild-type, *rcsD*, *rcsC* and *rcsD rcsC* strains; and compared with control without sucrose (empty bars). These assays were performed as described in Material and Methods. The error bars correspond to the standard deviation of three independent experiments done in duplicate.

In a previous study, we have demonstrated that when the threshold concentration of RcsB regulator has been reached the transcription of *rcsD* is suppressed, leading to a negative autoregulation mechanism of the Rcs system in *S.* Typhimurium [17]. Moreover, similar repression of *rcsD* was observed when other Rcs system activation conditions, like *rcsC11* and polymyxin B, were used [9], [17]. These results indicate that overexpression of *rcsB* could simulate the physiological changes that occur within the bacterial cell produced by other Rcs system-induction conditions. In addition, the *rcsC11* and *igaA* mutants produce a permanent activation of the Rcs system even in the absence of the environmental signals [7], [28]. Similar results were previously observed by Castanie-Cornet *et al*. when the Rcs phosphorelay system was activated by the expression of *djlA* or by overproduction of RcsB [29]. In agreement with these data, has been reported for other regulatory systems that overproduction of the response regulator mimics the physiological phosphorylation response [30]–[32]. On the basis of this finding, we investigated whether the expression of *rcsC* and *rcsD* genes are essential for the signaling transduction system under high concentrations of RcsB. For this purpose, we analyzed the expression levels of the well characterized RcsB-dependent *cps* operon in the absence of *rcsC* or *rcsD* genes and in the *rcsC rcsD* double mutant, overexpressing *rcsB* from p*rcsB* plasmid. In this assay we used the strain *cps*::MudJ harboring the *lacZ* chromosomal transcriptional fusion to *cps* operon. The negative controls were achieved using the above strains harboring the p*rcsB*op plasmid and growing in same condition. As expected, high levels of *cps* transcription were observed in the wild-type background containing p*rcsB*, compared with the negative control (Fig. 1A). Interestingly, *rcsC* and *rcsD* mutants displayed similar *cps* expression pattern, while in the *rcsC rcsD* double mutant no *cps* transcription was observed (Fig. 1A). To determine whether the RcsC and RcsD contribute to the modulation of other Rcs-dependent genes, we investigated the expression of *flhDC*::MudJ transcriptional fusion, an RcsB-repressed operon [11]. As shown in the Figure 1B we observed a remarkable decrease of *flhDC* expression when *rcsB* was overexpressed in the wild-type, *rcsC* and *rcsD* backgrounds, while no essential changes were observed in the *rcsC rcsD* double mutant compared with the control (Fig. 1B). Moreover, similar analyses were carried out using others RcsB-dependent genes like *wzz_st_* and *ugd*, obtaining similar modulation patterns than *cps* operon (data not shown). Here we note that all complemented mutants showed same expression patterns of reporter genes than the observed in the wild-type background (data not shown). In addition, when the Rcs system was activated by osmotic shock same *cps* and *flhDC* expression pattern was obtained in the analyzed strains (Fig. 1C and D, see Material and Methods). The same effect was observed when the strains were grown at low pH as activation condition (data not shown).

These results suggest that the RcsB-dependent genes modulation takes place only in the presence of RcsC and/or RcsD and at high concentration of the RcsB regulator. It is interesting to note that in the *cps* and *flhDC* expression analysis, the modulations levels were higher in the *rcsD* background than the values observed in the wild-type and *rcsC*. These finding allows us to postulate that: i) more than one signaling pathway can control the Rcs system activation, in which both RcsC or RcsD are able to act as histidine kinase proteins; and ii) the RcsC sensor is more effective in the phospho-transduction pathway.

### Effect of Acetyl-phosphate on the Rcs Phosphorelay Pathway

**Figure 2:**
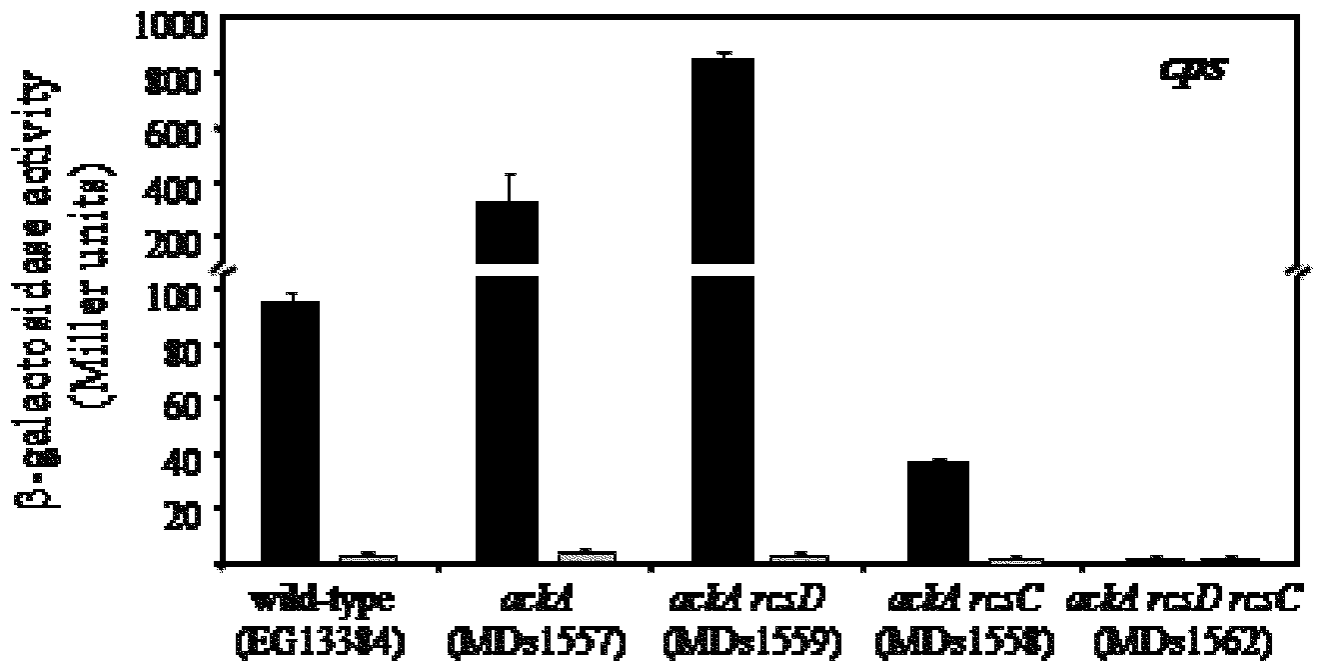
Effect of acetyl phosphate on *cps* expression in RcsC or RcsD-deficient cells. The transcriptional activity of *cps*::MudJ fusion, measured as β-galactosidase activity (Miller units), was investigated in five genetic backgrounds: wild-type (EG13384), *ackA* (MDs1557), *ackA rcsD* (MDs1559), *ackA rcsC* (MDs1558) and *ackA rcsD rcsC* (MDs1562) strains, carrying the p*rcsB* (black bars) or p*rcsB*op (grey bars) plasmids. The strains were grown at 37°C in LB medium supplemented with 0.35 mM as described in Material and Methods. The error bars correspond to the standard deviation of three independent experiments done in duplicate.

Since, the capsular and flagellar genes expression are affected by the acetyl-phosphate accumulation produced in the *ackA* mutant mediated by RcsB in the absence of RcsC [16], we decided to study the effect of acetyl-phosphate in the Rcs system when RcsB is overexpressed. To this end, we examined the expression of *cps*::MudJ in the *ackA rcsD*, *ackA rcsC* and *ackA rcsC rcsD* backgrounds carrying the p*rcsB* plasmid. As shown in Figure 2, the transcription levels of *cps* were 8-fold higher in the *ackA rcsD* mutant under *rcsB* overexpression than those obtained in the wild-type context. Moreover, we found that RcsB overproduction resulted in lower levels of *cps* expression in the *ackA rcsC* mutant compared with the wild-type levels, while in the the *ackA rcsD rcsC* mutant no *cps* transcription was observed (Fig. 2). Similar modulation patterns were obtained when the expression of the RcsB-dependent *flhDC* operon was analyzed (data not shown). It is important to note that no *cps* expression changes were observed in the tested background harboring the p*rcsB*op plasmid used as negative control (Fig. 2).

These results demonstrate that the acetyl-phosphate is accumulated in the *ackA* mutant at levels able to modulate the expression of *cps* or *flhDC* in an RcsB-dependent manner only when RcsC and/or RcsD are present. Furthermore, these data also suggest that in this condition RcsC is more efficient than RcsD in the transfer of the phosphate group directly to RcsB, because the highest *cps* expression was observed when only the RcsC sensor was maintained (Fig. 2, see *ackA rcsD* mutant).

### Analysis of the Interaction between RcsC, RcsD and RcsB

**Figure 3:**
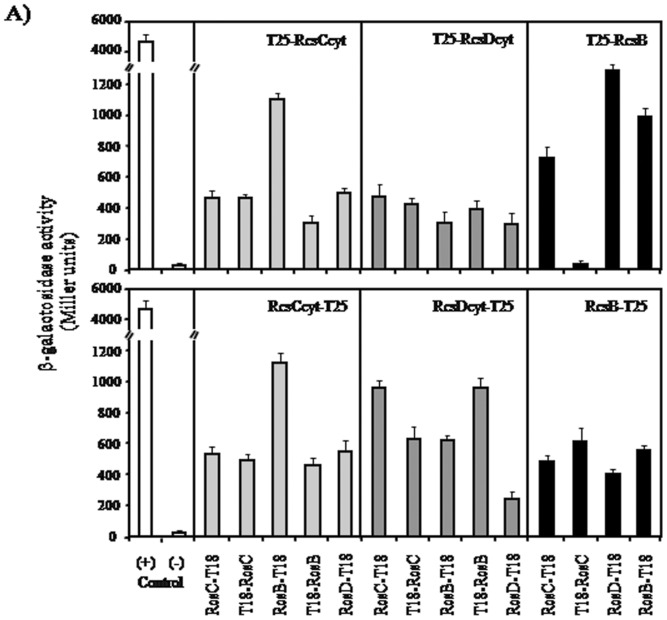
*In vivo* interaction analysis of the Rcs components: Bacterial two-hybrid assay. The ß-galactosidase activity expressed by *E. coli* DHM1 harboring derivatives plasmids encoding the *rcsC*cyt, *rcsD*cyt or *rcsB* genes fused to C-terminal (top panel) or N-terminal (bottom panel) of T25 fragments (in the top of each box) and these genes fused to N-terminal or C-terminal of the T18 fragment (under each bar), was measured from bacteria growing in stationary phase in LB medium containing 0.5 mM IPTG. Co-transformed cells with empty pKT25 and pUT18 vector plasmids producing basal ß-galactosidase activity levels served as negative control (−). Co-transformed cells with pKT25-zip and pUT18C-zip plasmid gave high ß-galactosidase activity levels served as positive control (+). The error bars correspond to the standard deviation of three independent experiments done in duplicate.

The results obtained to this point indicate that RcsB regulator can be activated by other pathways than the reported by Takeda *et al*. [3], where only the presence of RcsC or RcsD is necessary. These results suggest that both RcsC and RcsD should be able to interact with RcsB and with itself. To investigate these potential protein-protein interactions we used the bacterial two-hybrid (BACTH) assay developed by Karimova *et al*. [23]. In the Figure 3A we showed the results of such Rcs system component interactions, which were quantified as β-galactosidase activities. We observed that RcsB is a self-interacting protein, since the β-galactosidase values produced in strains with plasmids containing *rcsB* fused to the T18 or T25 fragments were 12 to 25 fold higher than the negative control. Our results of the self-interaction of RcsB are in accordance with the previously suggested by Majdalani and Gottesman [1].

Interestingly, the BACTH assays using RcsC or RcsD fused to the N- or C-terminal domain of T18 or T25 fragments provides evidence that both proteins were also able to interact with themselves. Here, we observed that the β-galactosidase values of RcsC-RcsC and RcsD-RcsD interactions were 12 and 5 fold higher than negative control, respectively (Fig. 3A). As expected, the data of this assay indicated that also RcsC and RcsD could interact, resulting in 10 to 22 fold increased values compared to the negative control (Fig. 3A). Furthermore, we found that RcsC and RcsD interact with RcsB, producing an increase of 6 to 22 and 6 to 19 fold β-galactosidase levels compared to the negative control, respectively (Fig. 3A). These findings would support our assumption that these membrane proteins could independently phosphorylate the RcsB regulator. We also verified the interactions between the Rcs system components by a glutaraldehyde cross-linking assay using the purified cytosolic domains of RcsC and RcsD (data not shown).

Collectively, the BACTH and cross-linking analysis provide evidence that support our hypothesis that RcsC and RcsD are self-interacting proteins able to activate the RcsB regulator. Here, we suggest that the RcsB phosphorylation can be mediated by RcsC-RcsC (homodimer) or RcsD-RcsD (homodimer) interaction.

### Purification of RcsC and RcsD Cytoplasmic HK Domains

In the majority of known sensors, involved in signal transductions by phosphorylation mechanisms, the autophosphorylation and trans-phosphorylation processes occur in their cytoplasmic domain [33], [34]. For this reason, we decided to purify the cytoplasmic domain of RcsC and RcsD in order to study the *in vitro* Rcs phosphorylation. The DNA fragments corresponding to the coding sequence of the RcsC and RcsD cytoplasmic domain were amplified from the *S.* Typhimurium 14028s genome and cloned into the pT7-7 vector. To improve the affinity chromatography purification, the amino terminus His_6_-tag was added to each protein coding sequence. The derivative pT*rcsC*cyt-His_6_ and pT*rcsD*cyt-His_6_ plasmids were controlled by DNA sequencing and no errors in the coding sequence of each domain were found. Moreover, we observed that the RcsC and RcsD cytoplasmic regions were able to induce the RcsB–dependent mucoid phenotype using the *in vivo* functional analysis (complementation assays, data not shown).

According to the above results we performed the purification assay by which the pT*rcsC*cyt-His_6_ and pT*rcsD*cyt-His_6_ plasmids were introduced into *E. coli* BL21 DE3 *rcsDBC* mutant, in order to avoid *E. coli* Rcs proteins contamination. The maximum level of expression was assayed using different IPTG concentrations and times for induction. We observed that the best expression levels were obtained using 0.1 mM IPTG and further incubation at 30°C for 3 h (data not shown). The protein cytoplasmic domains were purified under native conditions by Ni^2+^-NTA affinity chromatography following the protocol detailed in Material and Methods. The SDS-PAGE analysis confirmed the purity of this cytoplasmic domains, observed as a single band in the soluble purification fraction that correspond to the molecular weight expected to RcsCcyt and RcsDcyt (data not shown). In addition, we observed that these proteins were maintained stably along stored time.

### Autophosphorylation of RcsC and RcsD Cytoplasmic HK Domains

**Figure 4:**
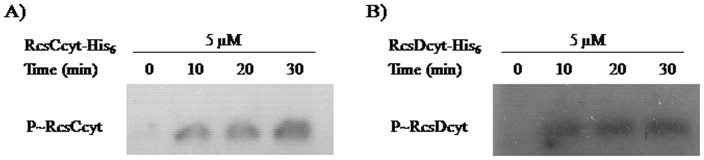
Autophosphorylation of RcsC and RcsD. The RcsCcyt-His_6_ (**A**) or RcsDcyt-His_6_ (**B**) proteins were incubated with [γ-^32^P] ATP as described in Material and Methods. The phosphorylation was stopped at different time points (0 to 30 min) by addition of 4× SDS sample buffer, and subjected to electrophoresis analysis in a 12% SDS-PAGE and them exposed to autoradiography.

To verify the capability of RcsC cytoplasmic domain (RcsCcyt-His_6_) to be autophosphorylated, it was incubated with radioactive ATP (see Material and Methods). As shown in Figure 4A, a detectable radioactive signal in the gel, corresponding to the molecular weight expected for RcsCcyt-His_6_, revealed a self-phosphorylation activity. We observed that the autophosphorylation was time-dependent reaching maximum level after 30 min of incubation, the last time assayed (Fig. 4A).

We also studied whether the RcsDcyt-His_6_ is a protein with self-phosphorylation activity, even when was described as unorthodox hybrid sensor lacking the conserved H residue in the putative kinase domain [1]. Interestingly, incubation of RcsDcyt-His_6_ with [γ-^32^P] ATP resulted in the appearance of a radioactive label band corresponding with the expected molecular weight (Fig. 4B). At difference of RcsCcyt-His_6_, the autophosphorylation signal of RcsDcyt-His_6_ took place at 10 min of incubation and the phosphorylate level was not increased in prolonged times (Fig. 4B). This result suggests that RcsD can works as a histidine kinase, supporting our hypothesis where we postulated that this protein might be involved in a new Rcs transduction mechanism where the RcsC kinase sensor is not required.

### Phosphate Group Transfer to RcsB

**Figure 5:**
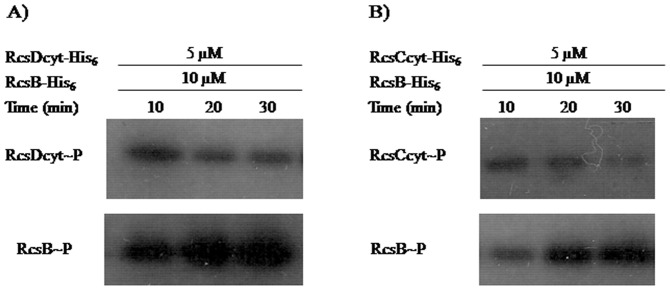
RcsC or RcsD catalyze phosphorylation of RcsB. The RcsB regulator was mixed with γ-^32^P-RcsDcyt-His_6_ (**A**) or γ-^32^P-RcsCcyt-His_6_ (**B**) as described in Material and Methods. The reaction was stopped at indicated times (10 to 30 min) by adding 4× SDS sample buffer, followed by analysis on 12% SDS-PAGE and exposed to autoradiography.

To test the phosphorylation of RcsB through RcsC or RcsD histidine kinase activities, we performed an *in vitro* trans-phosphorylation assay. For this purpose, the previously self-phosphorylated [γ-^32^P]-RcsCcyt-His_6_ or [γ-^32^P]-RcsDcyt-His_6_ was incubated with the purified RcsB response regulator as detailed in Material and Methods. To discard any RcsB autophosphorylation activity we performed a negative control incubating RcsB with [γ-^32^P]-ATP in the absence of RcsC or RcsD. In agreement with previous results [3], after that the RcsB protein was mixed with [γ-^32^P]-RcsDcyt-His_6_ the phosphate group was rapidly transferred to the RcsB regulator, resulting in a radioactive signal with the RcsB molecular weight (Fig. 5A). This radioactive mark was not observed when RcsB was alone incubated with [γ-^32^P]-ATP (data not shown). As expected, we noticed a decreased of the RcsDcyt label at the time that RcsB acquired the radioactive phosphate mark (Fig. 5A). Moreover, we also observed high trans-phosphorylation levels between RcsD and RcsB, suggesting that there is a strong affinity between them (Fig. 5A).

In order to explore the possibility that RcsB could be the phosphorylation target of RcsC, we exposed the purified RcsB protein with [γ-^32^P]-RcsCcyt-His_6_. As shown in Figure 5B, the presence of a radioactive band of equal RcsB molecular weight revealed that a trans-phosphorylation occurs between RcsC and RcsB proteins even in the absence of RcsD. We also observed that RcsCcyt lost its label during incubation with RcsB (Fig. 5B). These findings confirm our supposition about the presence of alternative transduction mechanisms for the Rcs system.

In these assays, we also incubated [γ-^32^P]-RcsCcyt-His_6_ or [γ-^32^P]-RcsDcyt-His_6_ with PmrA, the response regulator of the PmrAB system, in order to discard unspecific trans-phosphorylation of RcsC or RcsD. We observed that no radioactive band was produced (data not shown). This finding suggests that RcsC and RcsD are specific kinases to produce the RcsB phosphorylation.

In conclusion, these results demonstrated by the first time that RcsC promotes the phosphorylated state of RcsB independently of RcsD protein, showing that Rcs system implied a very specialized signal transduction mechanism that allows bacteria to respond at different environmental conditions.

## Discussion

In comparison with other known two component systems, the Rcs phosphorelay present several important differences, positioning it as a complex phosphorelay system. These differences mainly include the participation of two inner membrane proteins involved in the RcsB phosphorylation, the uncommon distribution of phosphate binding domains in these proteins and the wide variety of condition able to induce this system. The above features increase the possibilities that more than one signal initiates the phosphorelay cascade and affect the phosphate flow to RcsB. In this work, we further studied the Rcs system activation mechanism. Our results demonstrated, for the first time, that RcsC and RcsD can work independently as histidine kinase to transfer the phosphate group to RcsB.

Previously, we found that under certain condition *rcsB* is independent transcribed of the *rcsD* gene [9]. Moreover, we reported that the Rcs system is negatively autoregulated by high levels of RcsB regulator, repressing the transcription of *rcsD* [17]. These findings suggest us that in some cases the signaling pathways are independent of RcsD. In the same direction, evidence reported by Fredericks *et al.* showed that modulation of RcsB-dependent promoters occurs in an RcsC-independent manner [16]. These results allowed us to hypothesize that more than one signaling pathway may exist, in which only RcsC or RcsD are necessary. Supporting this hypothesis, we demonstrated that the expression of both *cps* and *flhDC* operons were modulated under high RcsB levels in an *rcsC* or *rcsD* mutant. These findings suggest that only the RcsB phosphorylated form promote modulation of gene expression, because no transcription changes were produced in the *rcsC rcsD* double mutant. Moreover, we observed a strong reporter gene modulation in the *rcsD* mutant. This fact could be explained assuming that no *rcsD* repression occurs in this mutant by RcsB, or that the retained RcsC protein is the most efficient sensor of the system.

On the other hand, we could not exclude that in absence of RcsC or RcsD the RcsB regulator is phosphorylated by a phosphodonor such as the acetyl phosphate. To address this point, we studied the *cps* and *flhDC* operons modulation in *ackA* mutant producing acetyl phosphate accumulation. We observed similar expression patterns when the *cps* or *flhDC* expressions were induced by RcsB overproduction and acetyl phosphate accumulation, but in this last condition the levels were higher than when only *rcsB* was overexpressed (Fig. 1 and 2). Our results demonstrated that this gene modulation was maintained in the *rcsC* and *rcsD* mutants. According with this notion Fredericks *et al.* reported that in the absence of AckA protein the *cps* and *flhDC* operons expression was controlled in an RcsC-independent manner. These authors suggested that this effect is produced by the RcsB phosphorylation mediated only by the acetyl phosphate accumulation [16]. However, we observed that in *ackA rcsD rcsC* mutant the gene modulation was abolished, indicating that the acetyl phosphate requires of RcsD or RcsC presence to generate the RcsB phosphorylation. In addition, we note that the higher levels of gene modulation were only maintained when the *rcsD* gene was deleted (Fig. 1 and 2). This observation also suggests that RcsC is the most active histidine kinase protein and that it can use acetyl phosphate as donor to phosphorylate RcsB. Our findings demonstrated that, at least for analyzed RcsB-reporter genes, the phosphorylated-RcsB is the active form which is produced by RcsC or RcsD kinase activity but not by other proteins. In agreement, Majdalani and Gottesman does not discard the existence of other phosphorylation pathways to previously reported, if the RcsB activity depends on its phosphorylated state [1].

In the present work, the BACTH results revealed that both RcsC and RcsD have the ability to self-interact, suggesting that both could be able to autophosphorylate. In addition, we found that both proteins interact with the RcsB regulator. These findings indicate that at least two additional phosphorylation mechanisms lead to the Rcs system activation. We propose that one of them involves the RcsC autophosphorylation and the phospho-transfer to RcsB (RcsC→RcsB), and the second one implicates the RcsD autophosphorylation and transfer of the phosphate group to RcsB (RcsD→RcsB).

At present, have been conducted a couple of failed efforts to purify the RcsC and RcsD active proteins. In fact, the results of Aiso and Ohki revealed that the mRNAs from *rcsC* and *rcsD* genes were extremely unstable and rapidly degraded [35]. On the other hand, Yamamoto *et al.* failed to overexpress the RcsC truncated form and could not observed self-phosphorylation activity of RcsD protein [34]. However, in the present study, we achieved for the first time the expression and purification of the RcsC and RcsD cytoplasmic domains as stable molecules.

We observed significant autokinase activity of RcsCcyt demonstrating conclusively the ability of this sensor to self-phosphorylate. On the other hand, the RcsD protein is considered as unorthodox sensor because the conserved H residue of the kinase catalytic domain is missing [36]. However, the presence of autophosphorylated RcsDcyt indicated that this protein is able to be self-phosphorylated. Importantly, we observed that the overproduction of the truncated proteins, as well as their corresponding complete version, were able to complement the absence of chromosomal copy in the mutants *rcsD rcsC*, supporting the *in vitro* assays results. Moreover, our data suggested that the RcsD HK domain contains a non conserved H located near to the active site that could be used as the phospho-receptor group under a signal presence. These results support the model of RcsCDB signal transduction postulated here, however the residues involved in each phosphorylation pathway still remain unknown. In this sense, we are conducting experiments that focus on the study of the structure and characterization of both the RcsC and RcsD HK domains by punctual mutations, mainly of those H residues located next to the RcsD pseudo-histidine kinase domain.

Previous experiments revealed the phosphate transfer between RcsD-HPt domain and RcsB [3]. These authors carried out the experiments with an uncompleted RcsD cytoplasmic domain which was phosphorylated by the ArcB sensor [3]. However, in this work we demonstrated that RcsC or RcsD cytoplasmic domains are able to transfer directly the phosphate group to RcsB regulator by *in vitro* phosphotransfer assay. The RcsB phosphorylation induced by RcsD reveals that RcsD could also work as sensor rather than an auxiliary protein. Moreover, the large radioactivity observed in the phosphotransfer assay between RcsD and RcsB could result from the absence of phosphatase activity of RcsD, present in RcsC. Collectively, our findings suggest that both proteins act as histidine kinase sensor for the RcsCDB system activation under different environmental conditions. This assumption is supported by our results obtained in the *in vivo* assays.

**Figure 6:**
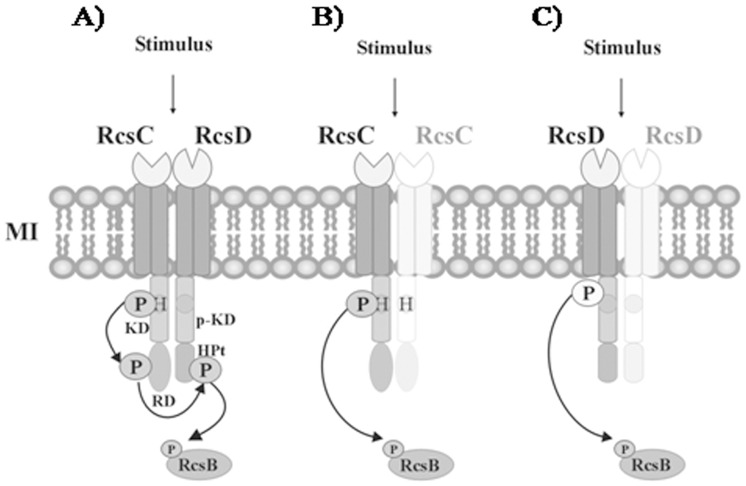
Signal transduction model of the RcsCDB phosphorelay system. In response to cell envelop stress, the Rcs system activation can proceed at least in three pathways: (**A**) After that an specific stimulus is sensed by RcsC, this protein is autophosphorylated and able to interacts with RcsD, leading to the transfer of phosphate group to the RcsB regulator. The RcsB phosphorylated form is able to bind the promoter region of those target genes required to produce an instant response. (**B**) Same or different stimulus can produce the RcsC autophosphorylation forming a complex with other no phosphorylated RcsC monomer, and then the phosphate group is transferred to RcsB to modulate the gene expression. (**C**) In different growth condition, other stimulus could be recognized by RcsD, which after autophosphorylation interacts with other no phosphorylated-RcsD monomer. Then, the phosphate group is transferred to RcsB in order to regulate the expression of those genes necessary for bacteria adaptation. H, histidine; P, phosphate group; KD, kinase domain; p-KD, pseudo kinase domain; RD, receiver domain; HPt, histidine phosphotransfer domain.

Taken together, these results allow us to postulate the following alternative phosphorylation pathways involved in the RcsB activation: i) After RcsC sense an specific stimulus it is autophosphorylated forming a complex with other no phosphorylated RcsC monomer, then the phosphate group is transferred to RcsB to modulate the gene expression (Fig. 6B); ii) under other environment, a different stimulus could be recognized by RcsD, which gets autophosphorylated and interacts with other RcsD monomer; then the phosphate is transferred to RcsB in order to regulate the gene expression (Fig. 6C). We propose that the different signals could be able to activate one of each alternative signaling pathway, producing different levels of the RcsB-phosphorylated form required to produce a differential gene modulation (Fig. 6). This differential gene modulation enables bacteria adapt at diverse environments. However, the mechanism of stimulus perception and the physiological signal remain to be elucidated. The complexity of the phosphorelay mechanisms raises the importance of this system on the bacterial response under a multiplicity of the medium changes. Further studies are currently in progress to explore which of the RcsB-regulated genes are controlled by the alternative mechanisms.
